# Efficacy and Cost-Effectiveness of MDM2 Fluorescence In Situ Hybridization (FISH) in Morphologically Atypical Spermatic Cord Lipomas

**DOI:** 10.7759/cureus.87852

**Published:** 2025-07-13

**Authors:** Zobash Noor, Shaymaa Hegazy, Rana Naous

**Affiliations:** 1 Pathology, University of Pittsburgh Medical Center, Pittsburgh, USA; 2 Pathology, University of Texas MD Anderson Cancer Center, Houston, USA

**Keywords:** atypical, lipoma, liposarcoma, mdm2, spermatic cord

## Abstract

Background: Spermatic cord lipomas originate from preperitoneal adipose tissue within the internal spermatic fascia and are found in 20%-70% of all inguinal hernia repairs. Morphologically, spermatic cord lipomas may harbor some atypical features, including increased stromal cellularity with thickened fibrous bands, occasional lipoblasts, and stromal nuclear atypia or hyperchromasia, raising concern for well-differentiated liposarcoma (WDLS) versus reactive-type atypia. Fluorescence in situ hybridization (FISH) for *MDM2* amplification would be the gold standard test in such cases. The aim of this study was to evaluate the efficacy and cost-effectiveness of *MDM2* FISH in diagnostically discrimination morphologically atypical spermatic cord lipoma cases at our institution.

Methods: All cases with “specimen type” labeled as “spermatic cord lipoma” between 2018 and 2023 were retrospectively retrieved from our institutional archives. Cases were included in the study group if they had *MDM2 *FISH performed. Cases with a prior history of spermatic cord well-differentiated liposarcoma with positive *MDM2* amplification were excluded. *MDM2* amplification status and corresponding final diagnosis were recorded.

Results: Three hundred twenty specimens labelled as spermatic cord lipoma were retrieved from our archives. Forty-three out of 320 had *MDM2* FISH performed. All 43 cases demonstrated atypical features and appeared morphologically similar. Only one out of 43 (2.3%) cases demonstrated a positive *MDM2* amplification result, thus labeling it as an incidental WDLS (11 cm) in the setting of an inguinal hernia repair for which the patient required a subsequent orchiectomy. The remaining 42 cases (97.7%) harbored reactive-type atypia with negative FISH results and were diagnosed as spermatic cord lipomas.

Conclusion: Our results indicate that 2.3% of morphologically atypical specimens labeled as spermatic cord lipoma at our institution are in fact well-differentiated liposarcomas. These findings prove the efficacy of *MDM2* FISH in diagnosing WDLS in the setting of atypical spermatic cord lipoma labeled specimens. Although 97.7% of our spermatic cord lipomas harbored reactive-type atypia with negative FISH results, which argues against the cost-effectiveness of such testing, we propose performing FISH analysis in cases greater than 10 cm in size to avoid misdiagnosis and ensure optimal patient care.

## Introduction

Spermatic cord lipomas originate from preperitoneal adipose tissue within the internal spermatic fascia and are found in 20%-70% of all inguinal hernia repairs [1]. Morphologically, spermatic cord lipomas may harbor some atypical features, including increased stromal cellularity with thickened fibrous bands, occasional pseudolipoblasts, and stromal nuclear atypia or hyperchromasia, raising concern for well-differentiated liposarcoma versus reactive-type atypia. Fluorescence in situ hybridization (FISH) for *MDM2* gene amplification would be the gold standard test [2] in such cases to help determine the nature of such atypical spermatic cord lipomas. The aim of this study was to evaluate the efficacy of *MDM2* FISH in diagnostically discriminating morphologically atypical spermatic cord lipoma cases at our institution, as well as assessing its cost-effectiveness by evaluating its clinical utility in comparison to testing burden.

## Materials and methods

All cases with “specimen type” labeled as “spermatic cord lipoma” between 2018 and 2023 were retrospectively retrieved from our institutional archives. Cases with a prior history of spermatic cord well-differentiated liposarcoma with positive *MDM2* amplification were excluded. Cases with atypical features, including increased stromal cellularity with thickened fibrous bands, presence of occasional lipoblasts, and stromal nuclear atypia or hyperchromasia that had *MDM2* FISH performed, were included in the study group. Patient age, specimen size, specimen laterality, *MDM2* gene amplification status, and corresponding final diagnosis were recorded.

For *MDM2* FISH testing, three 4-µm-thick tissue serial sections were cut from formalin-fixed paraffin-embedded (FFPE) tissue blocks for tissue adequacy evaluation and fluorescence in situ hybridization (FISH) studies analysis. The slides for the tissue adequacy assessment were stained with hematoxylin and eosin and reviewed by two pathologists. The hybridization was performed using commercially available dual-color probes: MDM2/SE12 12q15/12p11.1q11.1; MDM2 (orange spectrum) (Kreatech Biotechnologies, Amsterdam, Netherlands) and the SE12 (green spectrum) probes (Kreatech Biotechnologies, Amsterdam, Netherlands). *MDM2* FISH analysis was manually performed and quantitatively assessed by analysis of a minimum of 60 cells in the targeted region of interest circled by an expert pathologist along with a screen outside the region of interest. These areas are reviewed by at least two technologists with minimal discrepancy. Positive results of *MDM2* gene amplification by FISH are defined by a ratio gene/chromosome greater than two, while negative results are defined by a gene/chromosome ratio less than two.

## Results

Three hundred twenty spermatic cord lipoma specimens were retrieved. Forty-three out of 320 cases had atypical features corresponding to thickened fibrous septate with variable increase in cellularity, stromal nuclear atypia, and/or occasional pseudolipoblasts. The patient’s demographics including age, specimen laterality, size of the spermatic cord lipoma cases, and *MDM2* FISH amplification status are summarized in Table 1.

**Table 1:** Summary of patient’s demographics including age, specimen laterality, size of the spermatic cord lipoma cases, and MDM2 FISH amplification status. Case #: case number; age in years, size in centimeters (cm). FISH: fluorescence in situ hybridization.

Case #	Age	Laterality	Size (cm)	MDM2 FISH	Final Diagnosis
1	72	Left	5	Negative	Reactive atypia
2	59	Right	7.7	Negative	Reactive atypia
3	59	Right	20	Negative	Reactive atypia
5	73	Right	6.2	Negative	Reactive atypia
6	58	Right	6.3	Negative	Reactive atypia
7	61	Left	6.7	Negative	Reactive atypia
8	77	Left	6.5	Negative	Reactive atypia
9	63	Left	3.7	Negative	Reactive atypia
10	69	Left	14.8	Negative	Reactive atypia
11	46	Left	3.9	Negative	Reactive atypia
12	78	Right	6	Negative	Reactive atypia
13	58	Right	9.7	Negative	Reactive atypia
14	72	Right	29.2	Negative	Reactive atypia
15	47	Right	6.4	Negative	Reactive atypia
16	67	Bilateral	16	Negative	Reactive atypia
18	46	Left	10.8	Negative	Reactive atypia
19	66	Left	10.8	Negative	Reactive atypia
20	25	Left	5.2	Negative	Reactive atypia
21	63	Left	6.5	Negative	Reactive atypia
22	54	Left	6.7	Negative	Reactive atypia
23	81	Left	13.3	Negative	Reactive atypia
24	64	Left	5.7	Negative	Reactive atypia
25	87	Left	9.8	Negative	Reactive atypia
26	77	Left	6.5	Negative	Reactive atypia
27	57	Left	4.7	Negative	Reactive atypia
28	65	Left	5.5	Negative	Reactive atypia
29	65	Left	6.6	Negative	Reactive atypia
31	47	Right	7.6	Negative	Reactive atypia
32	63	Right	3.8	Negative	Reactive atypia
33	78	Left	7.2	Negative	Reactive atypia
34	67	Left	4.2	Negative	Reactive atypia
35	63	Right	3.8	Negative	Reactive atypia
36	63	Left	7	Negative	Reactive atypia
37	53	Left	8.6	Negative	Reactive atypia
38	69	Right	5.4	Negative	Reactive atypia
39	65	Left	6	Negative	Reactive atypia
40	45	Right	2.1	Negative	Reactive atypia
41	88	Right	6.5	Negative	Reactive atypia
42	63	Right	9.3	Negative	Reactive atypia
43	72	Right	11	Positive	Well-differentiated liposarcoma

**Figure 1:**
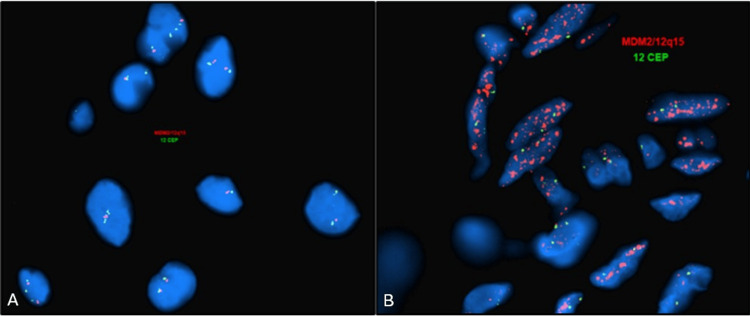
MDM2 FISH gene amplification results. A: Negative FISH for MDM2 gene amplification in an atypical spermatic cord lipoma case compatible with reactive atypia. B: Positive FISH for MDM2 gene amplification in an atypical spermatic cord lipoma case compatible with a diagnosis of well-differentiated liposarcoma. FISH: fluorescence in situ hybridization, CEP: Centromere Enumeration Probe.

All patients were identified as males per their medical record and presented with clinical and/or radiologic impression of inguinal hernia/cord lipoma. The results showed that one out of 43 (2.3 %) cases demonstrated *MDM2* amplification by FISH (Figures 1A, 1B) with a gene/chromosome ratio of 30.9 corresponding to a diagnosis of well-differentiated liposarcoma.

**Figure 2:**
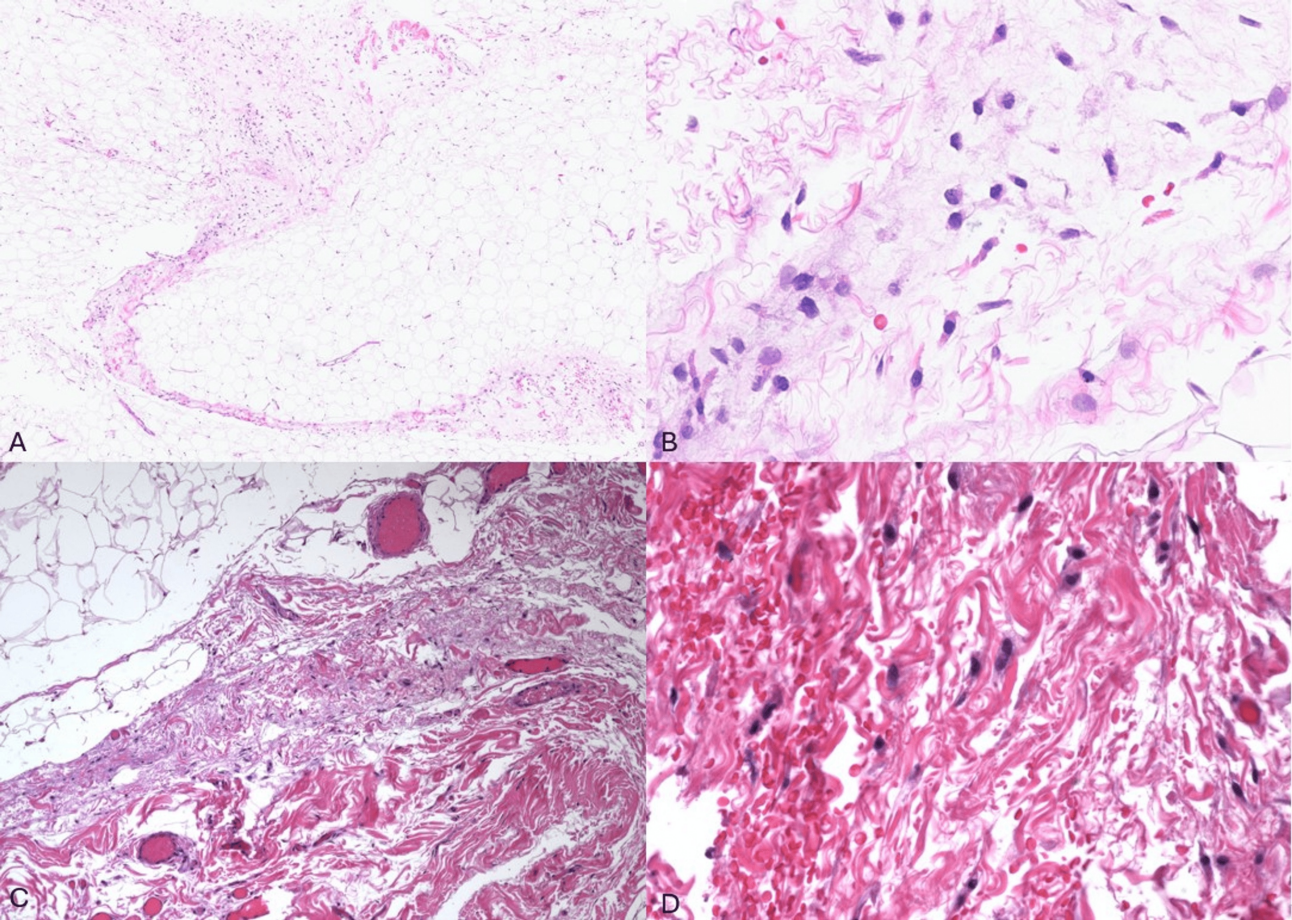
Similar histomorphologic features of atypical spermatic cord lipoma and well-differentiated liposarcoma. A-B: Atypical spermatic cord lipoma case demonstrating, on low- and high-power magnification, thickened fibrous septate with increase in cellularity, stromal nuclear atypia with variation in nuclear size, and occasional hyperchromasia (hematoxylin and eosin (H&E); 10× and 40×). C-D: Well-differentiated liposarcoma case demonstrating similar morphologic features to the atypical spermatic cord lipoma case with variably cellular fibrous septae and stromal atypia (H&E; 10× and 40×).

The patient was a 72-year-old man presenting for an inguinal hernia repair surgery. The size of his “lipoma of cord” specimen was 11 cm in maximum dimension (11 x 6 x 1.3 cm). Due to the diagnosis of well-differentiated liposarcoma in the setting of inguinal hernia repair, the patient subsequently underwent a right orchiectomy which showed a small focus of residual well-differentiated liposarcoma with no areas of dedifferentiation and negative margins (slides not available). Currently, the patient is undergoing close clinical follow-up with no recurrences to date. The remaining 42 (97.7%) cases, although had overlapping morphologic features with our well-differentiated liposarcoma case, were negative for *MDM2* gene amplification by FISH with a corresponding final diagnosis of spermatic cord lipomas with reactive-type atypia (Figures 2A-2D).

## Discussion

A lipoma of the cord is herniated fat that appears to originate from the preperitoneal fat outside and posterior to the internal spermatic fascia and protrudes through the internal ring lateral to the cord [1]. Although it is commonly found in inguinal hernia repair procedures, it is not always sent for histopathology evaluation. Lipomas may rarely undergo sarcomatous transformation and evolve into a liposarcoma. Liposarcomas are locally aggressive malignancies with a high incidence of local recurrence and variable prognosis depending on their dedifferentiation status and histologic grade [2]. Complete excision of liposarcomas of the cord is particularly important as local recurrence of these tumors is likely a consequence of incomplete excision with positive surgical resection margins, which can possibly promote tumor seeding through the operative site [3].

According to clinicopathological and molecular genetic characteristics, liposarcomas are grouped per the World Health Organization (WHO) into three categories, namely atypical lipomatous tumor or well-differentiated liposarcoma (ALT-WDLPS)/dedifferentiated liposarcomas, myxoid/round cell liposarcoma, and pleomorphic liposarcoma. Based on histology, a well-differentiated liposarcoma, in particular, the lipoma-like variant, can mimic fatty tissue or a benign lipoma [4]. In this study, we have studied the use of *MDM2* amplification by FISH to differentiate a benign lipoma of cord from well-differentiated liposarcoma, as this difference is crucial in prognosis and treatment. In lipomas of the spermatic cord, the presence of fibrous septa containing atypical cells with enlarged hyperchromatic nuclei can mimic a well-differentiated liposarcoma, and *MDM2* gene amplification by FISH can be a cost-effective and efficient method in such cases to avoid missing a well-differentiated liposarcoma of the spermatic cord.

The murine double minute (*MDM2*) oncogene is important in controlling the cell cycle by binding to TP53 and promoting its degradation. Well-differentiated and dedifferentiated liposarcomas share amplifications in the chromosomal region 12Q13-15. These amplifications constantly affect *MDM2* and can be detected by FISH [5]. Multiple studies have assessed the utility of immunohistochemical methods to evaluate for *MDM2* amplification [6]. Most of these studies have shown a lower sensitivity for immunohistochemical stains (45%) compared to FISH testing in assessing for *MDM2* gene amplification. In a study by Kashima et al., the sensitivity and specificity of *MDM2* amplification by FISH reached 93.5% and 100%, respectively [7]. Therefore, *MDM2* DNA amplification by FISH remains the gold standard for evaluating lipomatous tumors [8].

At the current time, there are no radiologic features that can reliably differentiate between atypical spermatic cord lipomas and well-differentiated liposarcomas, making histologic confirmation essential. Increased awareness and consideration of well-differentiated liposarcoma in the differential of atypical spermatic cord lipoma is essential when evaluating inguinal hernia repairs in order to facilitate accurate diagnosis and appropriate management.

During histological evaluation of a lipoma of spermatic cord specimen and using WHO criteria for assessing atypia in lipomatous lesions, increased stromal cellularity with thickened fibrous bands, occasional pseudolipoblasts, proliferation of spindle or epithelioid cells or stromal nuclear atypia or hyperchromasia can either be evidence of a disguised well-differentiated liposarcoma of cord or just a manifestation of benign reactive atypia. These reactive atypical histologies in a lipoma of cord are most commonly seen secondary to irritation in cases of an obstructed or incarcerated hernia or in long-standing hernias. Accurate recognition in such cases is essential to avoid misdiagnosis; however, it is worth noting that evaluation of such atypical features may be affected by interobserver variability assessment among pathologists. Fluorescence in situ hybridization (FISH) for *MDM2* gene amplification can be used to distinguish between benign reactive atypical cells and atypical lipomatous tumor/well-differentiated liposarcoma and would be the gold standard test in such cases [9,10].

Our one case out of 43 (2.3 %) spermatic cord lipoma cases that showed *MDM2 *gene amplification by FISH compatible with well-differentiated liposarcoma occurred in an older male, 72 years old, and had a size larger than 10 cm (11 cm in maximum dimension). Morphologically it showed similar features to the remaining 42 spermatic cord lipoma cases within our cohort and harbored some increase in stromal cellularity with thickened fibrous bands, some myxoid change, mild nuclear atypia with variation in nuclear size, and occasional hyperchromasia. There were no other distinguishing features that could set it apart from the remaining 42 FISH negative cases with no evidence of overtly and diffusely hyperchromatic stromal cells or true lipoblasts with hyperchromatic indented nuclei and cytoplasmic lipid vacuoles that are characteristically seen in well-differentiated liposarcomas. Review of our cohort revealed that eight out of the 43 cases had a specimen size larger than 10 cm with corresponding patient ages ranging from 46 to 81 years old. In a study by Clay et al., the authors recommended *MDM2* FISH testing for lipomatous cases with equivocal atypia, lipomatous lesions of the retroperitoneum/pelvis/abdomen, and deep extremity lesions that are >10 cm in patients over 50 years of age [11]. Extrapolating from these criteria, we propose performing *MDM2* FISH testing on atypical spermatic cord lipomas greater than 10 cm to avoid potential diagnostic pitfalls.

Although the one positive *MDM2* FISH result among our 43 cases may not be statistically significant, the value of *MDM2* FISH testing in morphologically atypical spermatic cord lipomas becomes more accentuated when comparing the minor FISH testing costs to the major costs of misdiagnosis leading to surgical re-operation or even delayed patient presentation with metastasis given that evaluation for *MDM2* gene amplification status is of therapeutic importance as amplified neoplasms are amenable to targeted therapy [12], and the patient would have missed the opportunity of targeted treatment. Additionally, since FISH testing has a low false negative rate with 93.5% sensitivity and 100% specificity for detecting *MDM2* gene amplification [7], this gold standard testing modality further emphasizes its reliability in diagnostically discriminating morphologically atypical spermatic cord lipoma specimens.

To our knowledge, there has not been any study in the literature regarding the use of *MDM2* gene amplification by FISH for differentiating atypical cells and increased stromal cellularity in lipomas of spermatic cord from well-differentiated liposarcoma. Although atypical spermatic cord lipoma can mimic well-differentiated liposarcoma, it can be confidently distinguished through FISH for *MDM2* gene amplification. Including well-differentiated liposarcoma in the differential diagnosis of atypical spermatic cord lipoma is important to ensure accurate diagnosis and prevent misdiagnosis.

One limitation in our study is the low sample size of 43 atypical spermatic cord lipomas. This may be attributed to the fact that the majority of spermatic cord lipomas in our practice and in general are commonly classic cord lipomas that are characterized by bland fibroadipose tissue and minimal to no atypical features. More studies with increased sample size are necessary to be able to reach definitive cutoff criteria for *MDM2* FISH testing on spermatic cord lipomas with atypical features.

## Conclusions

In conclusion, our results indicate that 2.3% of morphologically atypical specimens labeled as spermatic cord lipoma at our institution are in fact well-differentiated liposarcomas. Despite this low yield, these findings prove the efficacy of *MDM2* FISH in diagnosing well-differentiated liposarcoma in the setting of atypical spermatic cord lipoma specimens. Although 97.7% of our spermatic cord lipomas harbored reactive-type atypia with negative FISH results, which argues against the cost-effectiveness of such testing, we propose performing FISH analysis in cases greater than 10 cm in size to avoid misdiagnosis and ensure optimal patient care.
